# Rebounds of sevoflurane concentration during simulated trigger-free pediatric and adult anesthesia

**DOI:** 10.1186/s12871-023-02148-3

**Published:** 2023-06-08

**Authors:** Simon Zumsande, Christian Thoben, Nils Dennhardt, Terence Krauß, Robert Sümpelmann, Stefan Zimmermann, Henrik Rüffert, Sebastian Heiderich

**Affiliations:** 1 Clinic of Anesthesiology and Intensive Care Medicine, Hannover Medical School, Carl-Neuberg-Str. 1, 30625 Hannover, Germany; 2 Institute of Electrical Engineering and Measurement Technology, Department of Sensors and Measurement Technology, Leibniz University Hannover, Hannover, Germany; 3 Clinic of Anesthesiology and Intensive Care Medicine, Helios Klinik Schkeuditz, Leipzig, Germany

**Keywords:** Malignant hyperthermia, Trigger-free anesthesia, Patient safety, Volatile anesthetics, Washout method, Pediatric anesthesia, Rebound effect, Active charcoal filters

## Abstract

**Background:**

In trigger-free anesthesia a volatile anesthetic concentration of 5 parts per million (ppm) should not be exceeded. According to European Malignant Hyperthermia Group (EMHG) guideline, this may be achieved by removing the vapor, changing the anesthetic breathing circuit and renewing the soda lime canister followed by flushing with O_2_ or air for a workstation specific time. Reduction of the fresh gas flow (FGF) or stand-by modes are known to cause rebound effects. In this study, simulated trigger-free pediatric and adult ventilation was carried out on test lungs including ventilation maneuvers commonly used in clinical practice. The goal of this study was to evaluate whether rebounds of sevoflurane develop during trigger-free anesthesia.

**Methods:**

A Dräger® Primus® was contaminated with decreasing concentrations of sevoflurane for 120 min. Then, the machine was prepared for trigger-free anesthesia according to EMHG guideline by changing recommended parts and flushing the breathing circuits using 10 or 18 l⋅min^− 1^ FGF. The machine was neither switched off after preparation nor was FGF reduced. Simulated trigger-free ventilation was performed with volume-controlled ventilation (VCV) and pressure-controlled ventilation (PCV) including various ventilation maneuvers like pressure support ventilation (PSV), apnea, decreased lung compliance (DLC), recruitment maneuvers, prolonged expiration and manual ventilation (MV). A high-resolution ion mobility spectrometer with gas chromatographic pre-separation was used to measure sevoflurane in the ventilation gas mixture in a 20 s interval.

**Results:**

Immediately after start of simulated anesthesia, there was an initial peak of 11–18 ppm sevoflurane in all experiments. The concentration dropped below 5 ppm after 2–3 min during adult and 4–18 min during pediatric ventilation. Other rebounds of sevoflurane > 5 ppm occurred after apnea, DLC and PSV. MV resulted in a decrease of sevoflurane < 5 ppm within 1 min.

**Conclusion:**

This study shows that after guideline-compliant preparation for trigger-free ventilation anesthetic machines may develop rebounds of sevoflurane > 5 ppm during typical maneuvers used in clinical practice. The changes in rate and direction of internal gas flow during different ventilation modes and maneuvers are possible explanations. Therefore, manufacturers should provide machine-specific washout protocols or emphasize the use of active charcoal filters (ACF) for trigger-free anesthesia.

## Introduction

Malignant hyperthermia (MH) is a rare neuromuscular disorder, in which genetically predisposed individuals develop life-threatening metabolic crises [1] during general anesthesia caused by triggering substances like volatile anesthetics or succinylcholine. Patients with known or suspected increased risk of MH should therefore not be exposed to triggering substances. A trigger-free anesthesia is recommended instead. The Malignant Hyperthermia Association of the United States (MHAUS) [2] and the European Malignant Hyperthermia Group (EMHG) [3] both recommend that trace gas concentrations of any volatile anesthetic should not exceed 5 ppm during trigger-free anesthesia. But anesthetic machines continuously emit volatile anesthetics even after removal of the vaporizer because the substances are adsorbed and desorbed by rubber and plastic components inside the machine [4, 5]. Therefore, three different methods of preparation for trigger-free anesthesia are recommended by the EMHG and MHAUS: (1) Use of a dedicated machine that has never been contaminated with volatile anesthetics. (2) Use of active charcoal filters (ACF). (3) Washout method: Changing breathing circuits, soda lime canister and breathing system of the workstation with new uncontaminated components or autoclaved components and flushing the machine for a workstation specific time with O_2_ or air [3].

After preparation with the washout method the fresh gas flow (FGF) should not be reduced [6–9] and the machine should not be set to stand-by mode [6, 8, 10, 11]. In literature and expert opinion, it is widely assumed that after sufficient preparation of the anesthetic machines the concentration of volatile anesthetic would remain below 5 ppm during trigger-free anesthesia [7, 8, 12–14] but it was never tested. In this study, we prepared the anesthetic machines using the wash out method in accordance with EMHG guideline and subsequently simulated adult and pediatric trigger-free anesthesia including different ventilation maneuvers that are common in clinical practice. The goal of this laboratory study was to evaluate whether the anesthetic machine emits a sevoflurane concentration > 5 ppm during simulated trigger-free ventilation.

## Methods

### Contamination phase

**Fig. 1:**
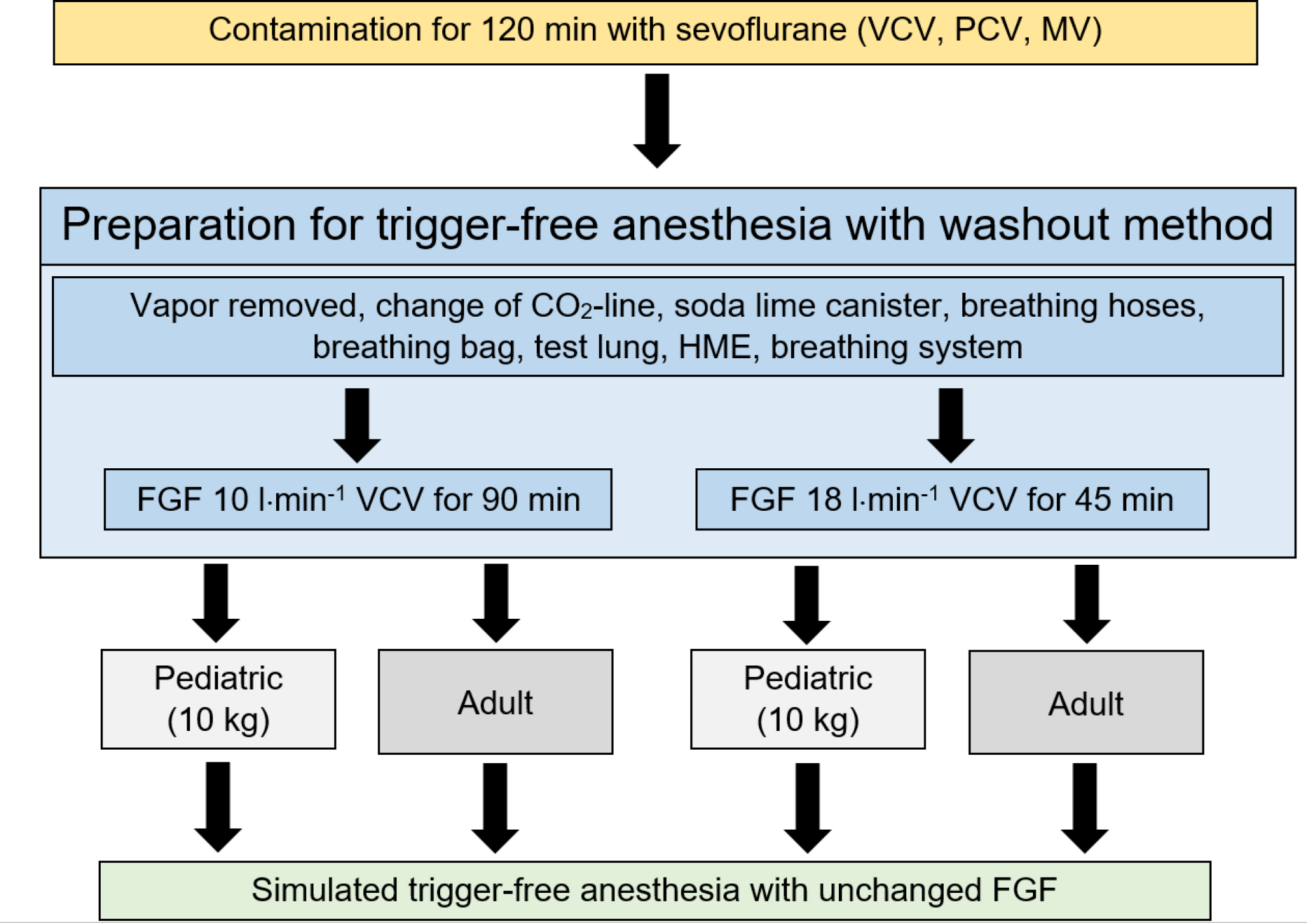
Study flow chart. *VCV*: volume controlled ventilation, *PCV*: pressure controlled ventilation, *MV*: manual ventilation, *FGF*: fresh gas flow, *HME* heat and moisture exchanger

A Dräger® Primus® was contaminated with sevoflurane for a total of 120 min as following: First, a test lung was ventilated with 8% sevoflurane using manual ventilation (MV) for 10 min in 4 l⋅min^− 1^ FGF, a tidal volume (VT) of 500 ml and a respiratory rate (RR) of 12 breaths min^− 1^. This was followed by 3% sevoflurane in 1 l⋅min^− 1^ FGF with continued MV for 20 min, pressure controlled ventilation (PCV) for 45 min and finally volume controlled ventilation (VCV) for 45 min. This contamination procedure was chosen to resemble the contamination during a typical course of a general anesthetic procedure with sevoflurane. Other settings during machine ventilation were: VT 500 ml, RR 12⋅breaths min^− 1^, positive end-expiratory pressure (PEEP) 5 mbar, inspired to expired time ratio (I:E) of 1:1.9. See Fig. 1 for an overview of the study protocol.

### Trigger-free preparation with washout method

After contamination the anesthetic machine was prepared for trigger-free anesthesia according to the EMHG guideline [3]: The vapor was removed and new, uncontaminated components (CO_2_-line, soda lime canister, breathing hoses, breathing bag, test lung) as well as freshly autoclaved components (breathing system) were assembled. Afterwards the machine was flushed for 45 min at 18 l⋅min^− 1^ FGF or 90 min at 10 l⋅min^− 1^ FGF as recommended in the EMHG guideline [3]. Other settings were: VCV, VT 500 ml, RR 12 breaths⋅min^− 1^, PEEP 5 mbar, I:E 1:1.9. At the end of the preparation process sevoflurane concentration was measured for benchmark recording.

### Adult protocol

A trigger-free anesthesia was simulated by ventilating a new test-lung using PCV, VCV, MV, and PSV:

First, the Y-piece of the breathing circuit was placed onto the circuit plug and the machine was set to spontaneous breathing mode with the adjustable pressure limiting valve (APL) open at 10 or 18 l⋅min^− 1^ FGF for 15 min respectively. This should simulate the time required for a patient to arrive in the operating theatre after preparation. Afterwards, the test lung was attached and the simulated ventilation started using PCV (VT 500 ml, RR 12 breaths⋅min^− 1^, PEEP 5 mbar, I:E 1:1.9) for 10 min. Various ventilation maneuvers followed: First, apnea phase for 2 min in spontaneous mode (apnea) to resemble breath-hold maneuvers needed during thoracic surgery or during magnet resonance scanning. Then, a manual recruitment maneuver was conducted. This was followed by simulating a decreased lung compliance (DLC) as seen in bronchospasm or insufficient depth of anesthesia. Therefore, a weight of 1600 g was put on the test lung and prolongation of expiration time. Between each ventilation maneuver the machine was set back to PCV for 2 min to normalize potential rebounds. Afterwards the protocol was repeated using the VCV mode with a pressure limit (Pmax) of 30 mbar starting at the 15 min interval simulating the wait for the patient’s arrival. To simulate the end of anesthesia with triggering of spontaneous breathing we used PSV with backup frequency of 3 breaths⋅min^− 1^ and a tidal volume of 250 ml for 5 min followed by manual ventilation with APL 12 mbar, RR 12 breaths⋅min^− 1^, VT 500 ml. Table 1 provides an overview and further information of the adult procedure protocol.

**Table 1:** Adult Experimental protocol: The timeline reads from top to bottom. APL adjustable pressure limiting valve, *FGF* fresh gas flow, *MV* manual ventilation, *VT* tidal volume, *PEEP* positive endexpiratory pressure, *RR* respiratory rate, *I:E* inspiratory:expiratory ratio, *Tinsp* inspiratory time, *PCV* pressure controlled ventilation, *PSV* pressure support ventilation, *VCV* volume controlled ventilation

	Maneuver	Time (min)	Machine mode	APL (mbar)	VT (ml)	PEEP (mbar)	RR (1⋅min^− 1^)	Tinsp (sec.)	I:E
**PCV**	Waiting for patient	15	Spont	0	0	0	0	-	-
	Normal ventilation	10	PCV	0	500	5	12	1.7	1:1.9
	Apnea	2	Spont	0	0	0	0	-	-
	Normal ventilation	2	PCV	0	500	5	12	1.7	1:1.9
	Recruitment	0.5	MV	30	1500	0	0	30	-
	Decreased lung compliance	3	PCV	0	200	5	12	1.7	1:1.9
	prolonged expiration	3	PCV	0	500	5	12	1.2	1:3.2
**VCV**	Waiting for patient	15	Spont	0	0	0	0	-	-
	Normal ventilation	10	VCV	0	500	5	12	1.7	1:1.9
	Apnea	2	Spont	0	0	5	0	0	-
	Normal ventilation	2	VCV	0	500	0	12	1.7	1:1.9
	Recruitment	0.5	MV	30	1500	5	0	30	-
	Decreased lung compliance	3	VCV	0	250	5	12	1.7	1:1.9
	Prolonged expiration	3	VCV	0	500	5	12	1.2	1:3.2
	Pressure support ventilation	5	PS	0	250	5	3	2.0	-
	Manual ventilation	2	MV	12	500	0	12	1.7	1:1.9

### Pediatric protocol

To simulate pediatric anesthesia of a 1-year-old 10 kg patient, the following changes were made compared to the adult protocol: at PCV and VCV, RR was set to 30 breaths⋅min^− 1^, the inspiratory time to 0.7 s, and VT to 70 ml (7 ml·kg^− 1^ body weight). The weight, which was put on top of the test lung during the simulated decreased lung compliance, was reduced to 540 g. No recruitment maneuver was simulated as this is not considered as good clinical practice in pediatric anesthesia. At PSV, the backup frequency was set to 10 breaths⋅min^− 1^ and VT was set to 35 ml to simulate induce spontaneous breathing attempts at the end of anesthesia. Concerning manual ventilation, VT was set on 100 ml and RR on 20 breaths min^− 1^. Table 2 shows details of the pediatric procedure protocol.

**Table 2:** Adult Experimental protocol: The timeline reads from top to bottom. APL adjustable pressure limiting valve, *FGF* fresh gas flow, *MV* manual ventilation, *VT* tidal volume, *PEEP* positive endexpiratory pressure, *RR* respiratory rate, *I:E* inspiratory:expiratory ratio, *Tinsp* inspiratory time, *PCV* pressure controlled ventilation, *PSV* pressure support ventilation, *VCV* volume controlled ventilation

	Maneuver	Time (min)	Machine mode	APL (mbar)	VT (ml)	PEEP (mbar)	RR (1⋅min^− 1^)	Tinsp (sec.)	I:E
**PCV**	Waiting for patient	15	Spont	0	0	0	0	-	-
	Normal ventilation	10	PCV	0	70	5	30	0.7	1:1.9
	Apnea	2	Spont	0	0	0	0	-	-
	Normal ventilation	2	PCV	0	70	5	12	0.7	1:1.9
	Decreased lung compliance	3	PCV	0	35	5	30	0.7	1:1.9
	Prolonged expiration	3	PCV	0	70	5	30	0.5	1:3
**VCV**	Waiting for patient	15	Spont	0	0	0	0	-	-
	Normal ventilation	10	VCV	0	70	5	30	0.7	1:1.9
	Apnea	2	Spont	0	0	0	0	0	-
	Normal ventilation	2	VCV	0	70	5	30	0.7	1:1.9
	Decreased lung compliance	3	VCV	0	35	5	30	0.7	1:1.9
	Prolonged expiration	3	VCV	0	70	5	30	0.5	1:3
	Pressure support ventilation	5	PS	0	35	5	10	2.0	-
	Manual ventilation	2	MV	12	100	0	20	1.0	1:2

### Detecting method

Sevoflurane concentration was detected using a high-resolution (resolving power of R_P_ = 90) ion mobility spectrometer (IMS) with gas chromatographic (GC) pre-separation, which was built at Leibniz University Hannover, and calibrated using a Vici® Dynacalibrator® Model 150 permeation oven and a gas mixing system for generating defined concentrations. The gas chromatographic pre-separation (GC temperature 50 °C, 10 m GC standard capillary column (Restek™, RTX^TM^-volatiles, inner diameter 530 μm, film thickness 2 μm)) separates a 10 µl gas sample into its components depending on their substance specific retention time. To increase sample rate, the method of multiple injections in a single experimental run (MISER) was applied to the GC pre-separation in which the subsequent sample is injected before all components of the previous injection elute from the separation column. This leads to the elution of the first analyte of the following chromatogram directly after the last analyte of the previous chromatogram [15–18]. Thereby, the sample frequency could be increased to 3 min^− 1^.

**Fig. 2:**
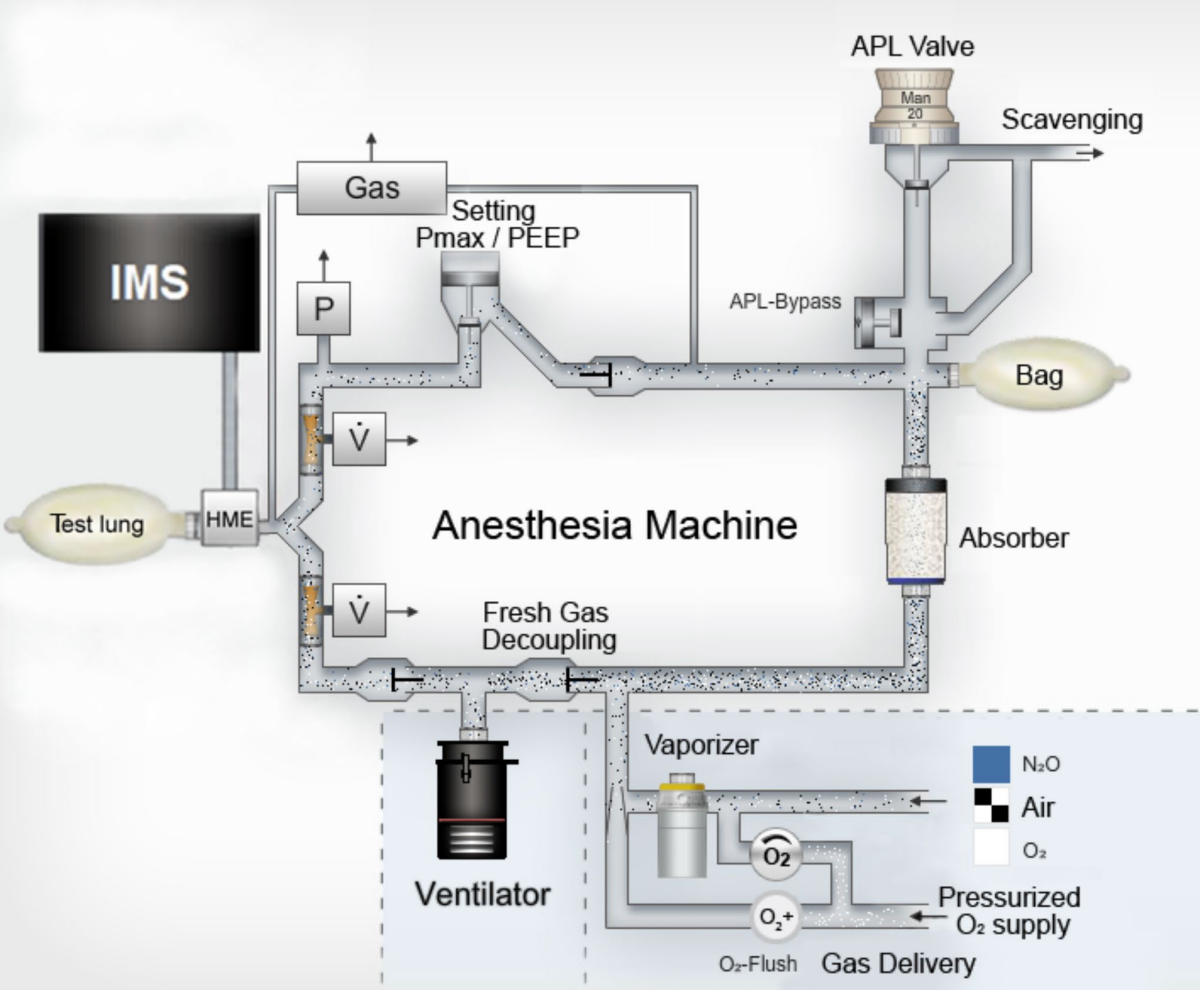
Experimental setup and gas flow diagram of Dräger® Primus®, modified from Drägerwerk AG & Co, Lübeck, Germany with kind permission. *APL* adjustable pressure limiting, *HME* heat and moisture exchanger, *PEEP* positive endexpiratory pressure, *pmax* maximum pressure, *V* gas flow sensor, *P* pressure sensor, *IMS* ion mobility spectrometer

After pre-separation the analyte (sevoflurane) is ionized in the IMS and injected into the drift region where the ions move in an electric field in a defined drift gas and are separated by their ion mobility. When the ionized analytes reach the detector, a current results depending on the amount of ionized analytes. Plotting the current over time gives the ion mobility spectrum. The IMS was developed to detect concentrations at the range of parts per trillion (ppt) [19]. Therefore, we added a dilution stage to dilute the sample in real time when the maximum capacity of die IMS is reached. Sevoflurane concentrations could be detected between 1 and 50 ppm at basic dilution settings. The standard deviation for concentrations at 5 ppm was ± 0.172 ppm. The sevoflurane sample was taken directly from the heat and moisture exchanger (HME) of the breathing circuits (Fig. 2).

## Results

At the end of the washout preparation sevoflurane concentration in all experiments was 1.476 ppm ± 0.784 ppm. Minimum and Maximum sevoflurane concentrations during different maneuvers are shown in Table 3.

**Table 3:** Sevoflurane concentration peaks during different ventilation maneuvers. *PCV* pressure controlled ventilation, *VCV* volume controlled ventilation, *ppm* parts per million, *Min.* minimal concentration, *Max.* maximal concentration, *n.a.* not applicable

	Maneuver	Sevoflurane concentration [ppm]								
		10 l⋅min^− 1^					18 l⋅min^− 1^			
		Adult		Pediatric			Adult		Pediatric	
		Min.	Max.	Min.	Max.		Min.	Max.	Min.	Max.
PCV	Normal ventilation	0	14.3	3.9	18.2		0	15.4	3.1	15.5
	Apnea	1.2	1.4	7.3	8.5		1.7	2.3	3.2	4.7
	Normal ventilation	1.7	4.4	7.4	7.7		2.5	5.9	4.1	5.8
	Recruitment	0.1	1.2		n.a.		0.1	2.0		n.a.
	Decreased lung compliance	1.2	3.0	7.2	9.0		2.3	3.5	4.6	5.2
	Prolonged expiration	1.1	3.2	4.0	7.2		1.2	3.3	3.2	6.0
VCV	Normal ventilation	0.2	11.1	0.1	15.1		0	13.7	3.2	12.5
	Apnea	1.2	1.7	3.8	4.3		1.3	1.6	3.3	4.3
	Normal ventilation	1.3	3.8	4.8	5.3		2.0	4.4	3.9	5,1
	Recruitment	0.1	0.1		n.a.		0.2	1,7		n.a.
	Decreased lung compliance	1.5	2.1	4.2	5.4		1.5	2.1	3.9	4.5
	Prolonged expiration	1.1	2.0	3.7	4.3		1.3	1.8	3.0	3,8
	Pressure support ventilation	1.2	4.5	3.9	6.2		1.3	4.5	2.9	5.8
	Manual ventilation	0.2	5.0	0.2	7.3		0.1	4.0	0.1	5.7

### Adult protocol

**Fig. 3:**
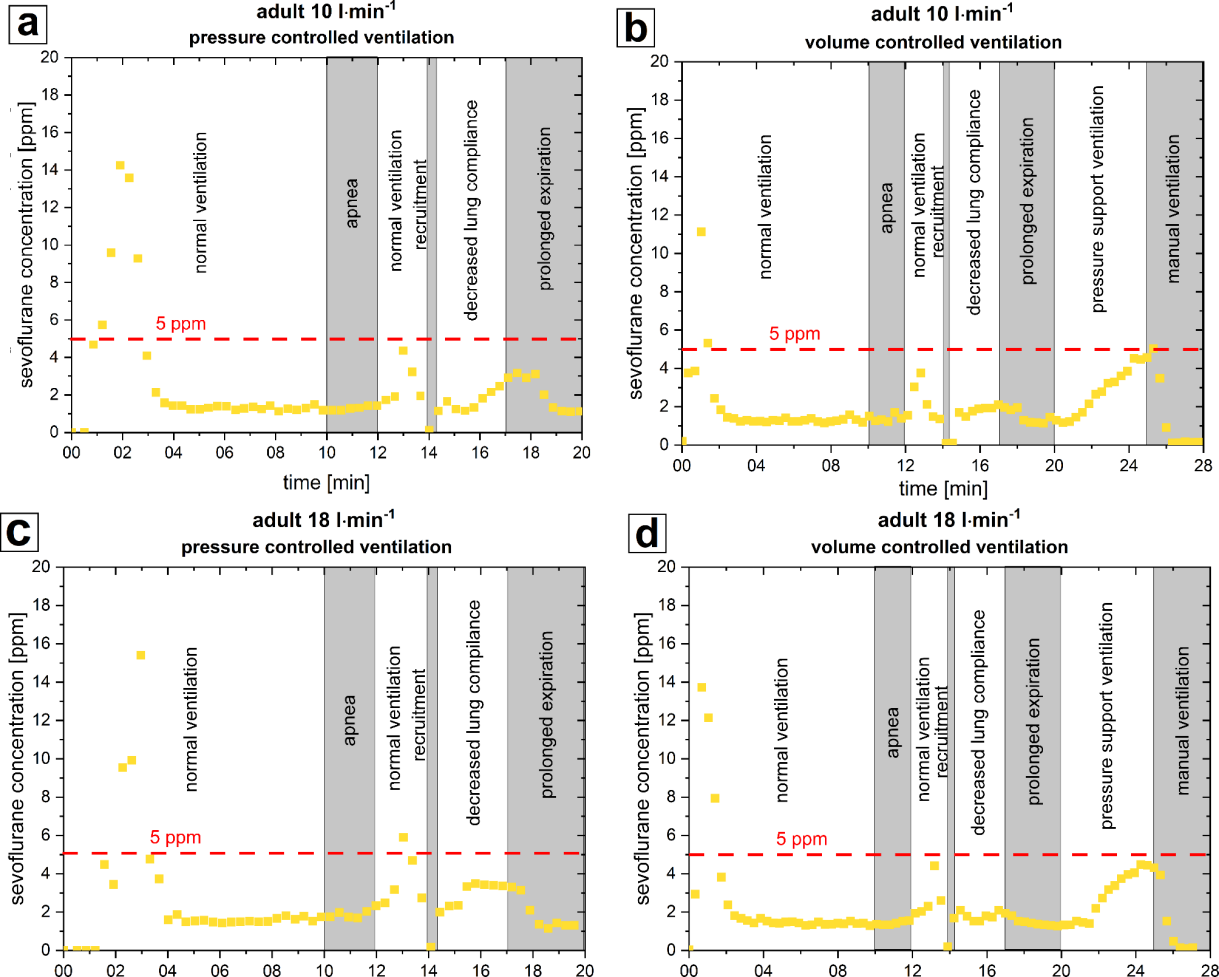
Results of adult experiments. *ppm* parts per million. **a**) 10 l·min^− 1^ fresh gas flow, pressure controlled ventilation **b**) 10 l·min^− 1^ fresh gas flow, volume controlled ventilation **c**) 18 l·min^− 1^ fresh gas flow, pressure controlled ventilation **d**) 18 l·min^− 1^ fresh gas flow, volume controlled ventilation

During the first minutes of trigger-free adult PCV the sevoflurane concentration showed a peak of 14 ppm (at 10 l⋅min^− 1^ FGF) and 15 ppm (at 18 l⋅min^− 1^ FGF) respectively. Afterwards the concentration dropped within 2–3 min below 5 ppm (see Fig. 3a, c). Peaks > 5 ppm also occurred during the first minutes of VCV as seen in Fig. 3b, d. During PSV a constant increase of sevoflurane concentration up to 4.5 ppm was noted without reaching a plateau within 5 min (see Fig. 3b, d). Manual Ventilation led to a sharp decrease in sevoflurane concentrations.

### Pediatric protocol

**Fig. 4:**
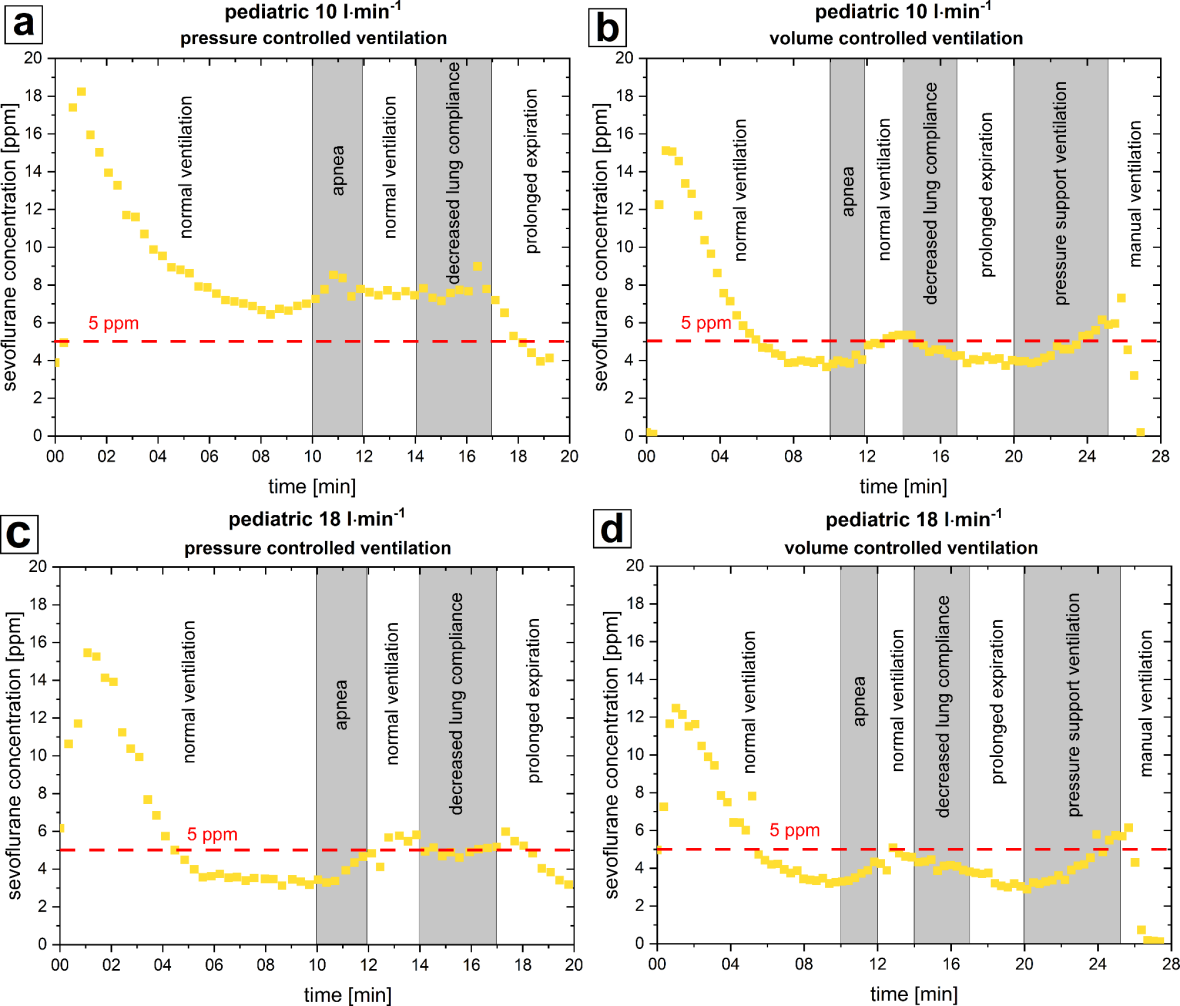
Result of pediatric experiments. *ppm* parts per million. **a**) 10 l·min^− 1^ fresh gas flow, pressure controlled ventilation **b**) 10 l·min^− 1^ fresh gas flow, volume controlled ventilation **c**) 18 l·min^− 1^ fresh gas flow, pressure controlled ventilation **d**) 18 l·min^− 1^ fresh gas flow, volume controlled ventilation

Within the first minute of trigger-free pediatric PCV the sevoflurane concentration showed a peak of 18 ppm (at 10 l⋅min^− 1^ FGF) and 15 ppm (at 18 l⋅min^− 1^ FGF). During the experiment the concentration dropped below 5 ppm at ventilation with 18 l⋅min^− 1^ FGF after 5 min but remained > 5 ppm for 18 min at ventilation with 10 l⋅min^− 1^ FGF (see Fig. 4a, c). Other peaks > 5 ppm were recorded during VCV and decreased lung capacity. During PSV sevoflurane increased to a maximum of 6 ppm and no plateau was reached within experimental time. Manual ventilation led to a sharp decrease of sevoflurane in all experiments (see Fig. 4b, d).

## Discussion

After preparation for trigger-free anesthesia using the washout method in accordance with EMHG recommendations the residual sevoflurane concentrations were 1.476 ppm ± 0.784 ppm. However, during simulated trigger-free anesthesia rebounds of sevoflurane > 5 ppm were detected during multiple ventilation modes and maneuvers, even though standby mode of the machine or FGF reduction were avoided after preparation. This can be explained by the different gas flow directions and rates inside the anesthetic machine resulting in different ventilation modes: The anesthetic machine used in this study operates a fresh gas decoupling valve to prevent dependency of tidal volume on FGF. Thereby the inspiratory part of the internal circuitry is flushed only intermittently during the respiratory cycle [8]. As a result, in our experiments the internal gas flow has been changed radically during simulated waiting for the patient: by placing the Y-piece of the breathing circuit onto the circuit plug, the FGF was directed through the absorber in direction of the scavenging system, leaving all parts from the fresh gas decoupling valve to the PEEP valve effectively unflushed (See Fig. 2). This may explain the initial rebounds observed. Other rebounds developed later during the simulated anesthesia. All rebound effects observed were higher during pediatric ventilation compared to adult ventilation and were detected even at maximum FGF (18 l⋅min^− 1^). Especially, the sevoflurane concentration in the pediatric 10 l⋅min^− 1^ FGF experiment was > 5 ppm during most of the experimental time.

Because of insufficient literature, EMHG and MHAUS guidelines do not differentiate between adult and pediatric trigger-free anesthesia. MHAUS generally recommends keeping the FGF at a minimum of 10 l⋅min^− 1^ at all times to avoid rebound effects. In this study the effort failed: even at 18 l⋅min^− 1^ FGF relevant sevoflurane rebounds occurred during simulated ventilation of a 10 kg pediatric patient. It should be highlighted, that a low FGF in pediatric anesthesia is strongly recommended along with breathing system filters to prevent loss of heat and airway moisture [20, 21], which leads to conflicting recommendations regarding trigger-free pediatric anesthesia.

### Clinical relevance of rebounds

Are the detected rebounds clinically relevant? Unfortunately, it is still unknown which residual concentration of volatile anesthetics is safe for MH suspected patients to breathe in. First, MH suspected individuals are a heterogenous group affected by different genetic causes: Until now, there are 50 known mutations in the RYR1 and CACNA1S genes accepted as diagnostic mutations by the EMHG. More gene loci are currently under investigation (STAC3) [22]. Second, there are epigenetic effects that are not fully understood: a male predominance of clinical MH crises and diagnosis after muscle biopsy was repeatedly reported [23–26], other factors include pre-operative exercise, pyrexia [27] and body mass index [28]. Therefore, threshold levels of volatile anesthetics are thought to be highly individual.

Still, acute MH is believed to be a dose depending process; therefore, it may be unlikely that a short exposition to volatile anesthetic trace gas concentrations in the low ppm range would cause an acute MH crisis. However, there are numerous case reports in which MH suspected patients developed severe MH-like reactions even in the absence of general anesthesia [29–31]. Until now, it is not understood which MH suspected patients are at risk of such episodes and consequently EMHG and MHAUS both chose < 5 ppm of any volatile anesthetic concentration in trigger-free anesthesia for safety reasons. Using this definition, the tested anesthetic machine did not meet the requirements for trigger-free ventilation after preparation with the EMHG guideline compliant washout method. Until more data is available, it should be assumed that other anesthetic machines by other manufactures might show similar effects.

## Limitations

There are some limitations to this study: First, the trigger-free anesthesia was performed on test lungs. Effects like heat, moisture, or emission of metabolic waste products from a patient were not simulated. Second, all experiments were performed on one sevoflurane contaminated anesthesia machine. Other machines of the same type may lead to different results, depending on long term contamination levels with volatile anesthetics, state of wear, age of plastic and rubber components. Third, all maneuvers during the simulated trigger-free anesthesia were performed in sequence during the experiment to resemble a real anesthetic procedure. Therefore, sevoflurane peaks during different maneuvers might be a result of a prior maneuver or ventilation mode.

## Conclusion

Despite successful removal of sevoflurane concentration below 5 ppm using the washout method in accordance with EMHG recommendations, the anesthetic machine was in fact not clean but emitted continuously small amounts of sevoflurane. This led to relevant rebounds during simulated trigger-free ventilation even at maximal FGF (18 l⋅min^− 1^). Common features of the ventilation maneuvers that led to rebounds were low minute ventilation and low VT as well as no ventilation such as during the waiting period at the beginning of the maneuvers. In the tested anesthetic machine, a fraction of the FGF is directed through the manual ventilation bag, even during mechanical ventilation. It can be assumed that the rebounds observed in this experiment may be higher in anesthetic machines that separate the manual ventilation part from the FGF during mechanical ventilation. In Summary, standby mode should always be avoided, and flushing the machine by ventilating a test lung with air or O_2_ should be continued until the patient arrives in the operating theatre. We further emphasize the necessity for manufacturers to provide machine specific washout instructions which should specifically designed to prevent rebounds of volatile anesthetics, as recommended for the instruction for use of an anesthetic workstation in the EN ISO Standard 80601-2-13:2022. In the absence of such specific protocols, we prefer the use of ACF or uncontaminated anesthesia machines instead of the washout method. In pediatric trigger-free anesthesia the washout method should not be used at all because a necessary low-flow ventilation for conservation of humidity and temperature leads to prolonged rebounds of volatile anesthetics. In those cases the use of an uncontaminated anesthesia machine or the use of ACF, which have shown to be safe even when using a FGF of 1 l⋅min^− 1^[10], seems to be superior compared to the washout method.

## Data Availability

The datasets used and/or analyzed during the current study are available from the corresponding author on reasonable request.

## Abbreviations

ACF: activated charcoal filters
APL: adjustable pressure limiting valve
DLC: decreased lung compliance
EMHG: European Malignant Hyperthermia Group
FGF: fresh gas flow
GC: gas chromatographic pre-separation
IMS: ion mobility spectrometer
I:E: inspired to expired time ratio
MAC: minimum alveolar concentration
MH: malignant hyperthermia
MHS: malignant hyperthermia susceptible
MHAUS: Malignant Hyperthermia Association of the United States
MISER: multiple injections in a single experimental run
MV: manual ventilation
PCV: pressure control ventilation
Pmax: maximum pressure
ppb: parts per billion
ppt: parts per trillion
PSV: pressure support ventilation
RR: respiration rate
Spont: spontaneous breathing mode
Tinsp: inspiratory time
Tplat: time of plateau pressure
VCV: volume controlled ventilation
VT: tidal volume
